# Influences of Hyperbranched Polyester Modification on the Crystallization Kinetics of Isotactic Polypropylene/Graphene Oxide Composites

**DOI:** 10.3390/polym11030433

**Published:** 2019-03-06

**Authors:** Zengheng Hao, Lu Li, Bo Yang, Xingyue Sheng, Xia Liao, Leilei He, Pan Liu

**Affiliations:** 1 Chongqing Zhixiang Paving Technology Engineering Co., Ltd., Chongqing 401336, China; haozengheng@cmhk.com (Z.H.); shengxingyue@cmhk.com (X.S.); heleilei@cmhk.com (L.H.); liupan@cmhk.com (P.L.); 2 College of Polymer Science and Engineering, State Key Laboratory of Polymer Materials Engineering, Sichuan University, Chengdu 610065, China; liaoxia@scu.edu.cn; 3 School of Civil Engineering, Chongqing Jiaotong University, Chongqing 400074, China

**Keywords:** isotactic polypropylene, graphene oxide, hyperbranched polyester, crystallization kinetics, nucleation and crystal growth

## Abstract

In this study, the hyperbranched polyester grafted graphene oxide (GO-H202) was synthesized, and the isotactic polypropylene/graphene oxide (iPP/GO) composites were prepared. Results of X-ray photoelectron spectra (XPS), Fourier transform infrared (FT-IR), and transmission electron microscopy (TEM) revealed the successful synthesis of GO-H202, while thermogravimetric analysis (TGA) indicated that the weight ratio of grafting was about 35 wt %. Differential scanning calorimetry (DSC) and polarized optical microscopy (POM) were carried out to investigate the role of GO and GO-H202 on the crystallization kinetics of the composites. Results suggested that the addition of GO enhanced the nucleation rate and crystallizability of the composites, while GO-H202 exhibited a higher crystallization acceleration effect compared to neat GO; results of isothermal crystallization kinetics and self-nucleation isothermal crystallization kinetics showed that both the overall crystallization rate and crystal growth rate increase after the addition of GO and GO-H202, and the crystallization acceleration of GO-H202 became evidently stronger compared to GO. Moreover, the variation trends of Avrami exponent *n* with the isothermal crystallization temperature *T_cISO_* changed significantly after the addition of GO or GO-H202, which might imply that the addition of GO and GO-H202 lead to different crystallization dimensionalities during the isothermal crystallization of the composites. The related mechanism was also discussed.

## 1. Introduction

Isotactic polypropylene (iPP) is one of the most widely used general-purpose plastics nowadays because of its low price and outstanding properties, such as its mechanical properties, chemical stability, nontoxicity, and easy processing [1,2,3,4,5,6]. iPP also exhibits interesting polymorphic behavior, which has many crystalline structures, including *α*- [2,7,8,9], *β*- [10,11,12,13], and *γ*-phase [14,15]. Among these crystal forms, the *α*-phase is the most stable and commonly seen crystalline form and can be obtained under practical processing conditions. *β*-Phase is a thermodynamically metastable crystalline form which can only be obtained under certain ways [16,17,18,19], such as crystallization in the melt shear field [18], in a temperature gradient field [20], or in the presence of *β*-nucleating agents [13,21,22]. To meet the increasing performance requirements of the products and to further increase the mechanical properties are some of the main challenges in the related area. 

Introduction of inorganic fillers has been found to be an efficient way to improve the strength of iPP without sacrificing other properties. Graphene is a two-dimensional atomic crystal composed of a single atomic layer of carbon atoms connected by *sp^2^* hybridization, which has a thickness of only 0.34 nm, known as the thinnest material [23]. This special structure provides graphene excellent properties such as outstanding electrical conductivity, thermal conductivity, high elastic modulus, larger specific surface area, and fractional quantum hall effect [24,25,26]. However, the strong Van der Waal force of graphene results in the characteristics of hydrophobicity and easy agglomeration, and the application of graphene is greatly limited [27]. Graphene oxide (GO) is a precursor of graphene, which has similar structure to graphene and can be used as the preferred choice for reinforcing polymers because of its carboxyl groups and carbonyl groups at the edge and hydroxyl groups and epoxides groups on the matrix [28,29]. The existence of these functional groups improves the hydrophilicity of GO, which can also react with polar polymers. For nonpolar polymers, functional groups are introduced to modify the surface of GO. Surface-functionalized GO exhibits good interfacial compatibility in nonpolar polymers such as iPP and polystyrene [13,30]. Composite modification of iPP with GO is an important way to make high-performance iPP materials.

Hyperbranched polymers have a lower branching efficiency than dendrimers while enjoying many useful properties [31,32,33,34]. The highly branched architecture minimizes chain–chain ability and low melt viscosity of these polymers [35], which is compatible with iPP. The presence of numerous functional groups (–OH) provides high reaction activity with inorganic filler, providing it the possibility to be desirable macromolecular surface modifier for GO to further enhance the compatibility and dispersion in iPP.

In this study, the GO grafted with hyperbranched polyester was prepared to enhance the interaction between GO and iPP matrix. Then, PP/GO composites were prepared to study the impacts of raw GO and hyperbranched polymer ester grafted GO on the detailed isothermal crystallization kinetics of the composites, which is an important issue that has not been reported before.

## 2. Experimental Section

### 2.1. Materials

**Scheme 1:**
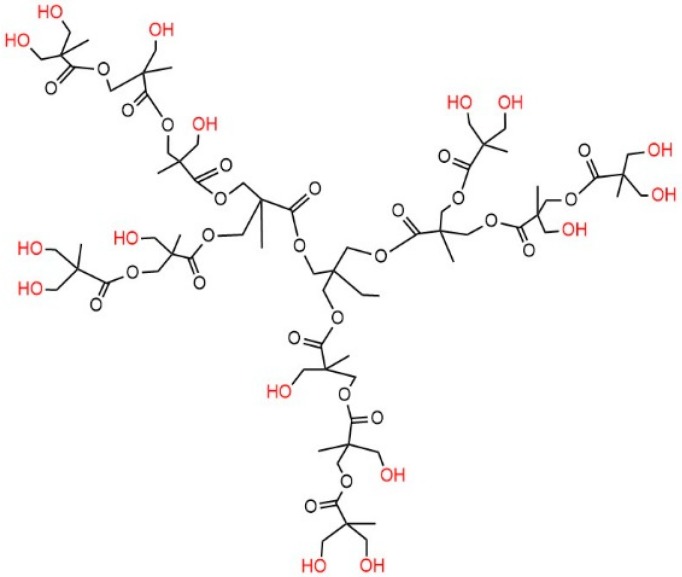
Chemical structure of the hyperbranched polyester H202.

iPP with a trade name of T38F, molecular weight of 347,200 and dispersion index of 3.63, average isotacticity of 97%, and melt index of 3.0 g/10 min (2.16 kg, 230 °C) was purchased from Lanzhou Petroleum Chemical Co., Ltd. (Lanzhou, China); Graphene oxide (GO) was purchased from Henan Angstron Graphene Technology Co., Ltd. (Zhengzhou, China); Hyperbranched polyester H202 (Scheme 1, *M_w_* = 1200 g/mol, hydroxyl number = 520 mg KOH/g polymer, denoted as Hyper H202 in this study) was purchased from Shanghai Seebio Biotech, Inc., (Shanghai, China) and used as received; *N*,*N*-dimethylformamide (DMF) was purchased from Sinopharm Chemical Reagent Beijing Co., Ltd. (Beijing, China), China, and was used as received.

### 2.2. Grafting of the Graphene Oxide

**Scheme 2:**
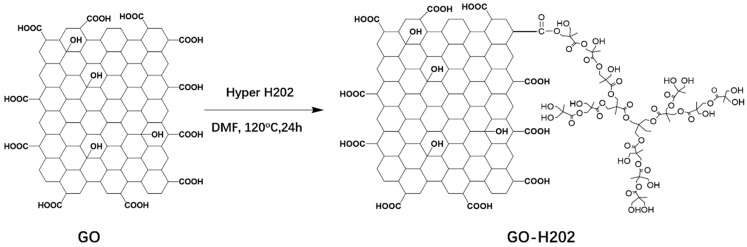
Reaction principle of hyperbranched polyester H202 modified graphene oxide (GO).

Reaction principle of hyperbranched polyester H202 modified graphene oxide is shown in Scheme 2. The hyperbranched polyester H202 and GO were dissolved in DMF at room temperature and then irradiated using ultrasound for 1 h [21,36]. Then, they were heated up to 120 °C and reacted under magnetic stirring for 24 h. After that, they were cooled to room temperature slowly and filtered using PVDF filtration membrane with an average pore diameter of 0.22 μm. The product was firstly washed by acetone and filtered several times to eliminate the unreacted H202 and extra solvent. Each time, the washed acetone was taken for FT-IR analysis, and the washing was finished until the washed acetone had the same FT-IR spectra compared with pure acetone. The collected material was then heated under vacuum drier at 80 °C for 24 h, and the final product, GO grafted with H202 (named as GO-H202), was obtained. The obtained raw GO and H202-grafted GO powder was vacuum dried at 60 °C for 24 h before use.

### 2.3. Preparation of iPP/GO Composites

The dried GO or GO-H202 powder was ultrasonically dispersed in a 200 mL xylene solution for 30 min. Then, a certain amount of iPP was added to the solution, keeping the mass fraction of GO or GO-H202 at 5%. After that, the solution was stirred at 130 °C for 2 h. Finally, the mixture was vacuum filtered and dried for 24 h to obtain iPP/GO and iPP/GO-H202 master batch. The iPP/GO master batch and neat iPP were mixed using twin-extruder to obtain the iPP/GO and iPP/GO-H202 composites, in which the concentration of GO or GO-H202 was 1 wt %. Then, a pressure-molding machine was used to press the samples. The neat iPP, iPP/GO, and iPP/GO-H202 were named as PP, GPP, and GHPP in this manuscript.

### 2.4. Characterization

#### 2.4.1. X-ray Photoelectron Spectra (XPS)

XPS was carried out by the use of an ESCALAB 250 photoelectron spectrometer (ThermoFisher Scientific, Waltham, MA, USA) with Al Kα (1486.6 eV) as the X-ray source set at 150 W and a pass energy of 30 eV for a high-resolution scan.

#### 2.4.2. Fourier Transform Infrared (FT-IR)

A Nicolet iS50 FT-IR spectrometer (Thermo Scientific, Waltham, MA, USA) was used to determine the FT-IR spectra of the samples. The FT-IR was recorded at a resolution of 4 cm^−1^, and 20 scans were averaged for each spectrum. The scanning range was 4000–400 cm^−1^ [37,38].

#### 2.4.3. Thermogravimetric Analysis (TGA)

TGA was carried out on a TA Q5000IR thermo-analyzer instrument (TA Instruments Inc., New Castle, DE, USA) from room temperature to 800 °C at a linear heating rate of 10 °C/min under N_2_ and air atmosphere (flow rate of 100 mL/min).

#### 2.4.4. Transmission Electron Microscopy (TEM)

To study the morphologies of the GO and GO-H202, transmission electron microscopy (TEM, Tecnai G2 F20, FEI, Hillsboro, OR, USA) was applied [39].

#### 2.4.5. Differential Scanning Calorimetry (DSC)

All the calorimetric experiments were carried out using a Mettler Toledo DSC3+ differential scanning calorimetry (DSC) under nitrogen atmosphere (50 mL/min). Calibration for the temperature scale was performed using indium as a standard to ensure reliability of the data obtained [40,41]. In order to ensure the homogeneity of samples and good contact between sample and pan, the virgin polymer was molded at 190 °C, 10 MPa for 5 mins into sheets of uniform thickness about 500 μm. Then, 3 mg round samples were punched out of the sheets.

The degree of crystallinity, *X*_c_, was calculated from the ratio *△H*_m_/(*△H*_u_ ▪ *W_f_*). *△H*_m_ and *△H*_u_ were the apparent and completely crystalline heats of fusion, respectively, and *W_f_* was the weight percentage of the polymer in the composites. *△H*_m_ was determined by linear interpolation of the baseline between the clear-cut end of the melting endotherm and its onset arbitrarily taken at 90 °C for all samples, and *△H*_u_ was 209 J/g for iPP [42,43,44].

#### 2.4.6. Isothermal Crystallization Kinetics

The isothermal crystallization studies were performed according to the following procedures [45,46,47]:

(a) Samples were heated from 50 to 200 °C at 10 ℃/min and kept for 5 min to erase any previous thermal history.
(b) Samples were cooled down to the desired crystallization temperature *T_cISO_* at 50 °C/min.
(c) Samples were isothermally kept for a certain time to complete crystallization.

#### 2.4.7. Self-Nucleation Isothermal Crystallization Kinetics

The self-nucleation isothermal crystallization kinetics was measured according to the manner proposed by Muller et al. [48,49]:

(a) The self-nucleating behavior of samples was firstly measured according to Fillon et al. [50,51,52], and the temperature region for the self-nucleation domain (Domain Ⅱ) was obtained.
(b) The samples were heated from 50 to 200 °C at 10 ℃/min and held for 5 min to destroy any residual nuclei.
(c) After that, samples were cooled to 50 °C at 10 °C/min and held for 2 min to create a “standard” thermal history [53], and then they were heated to the self-nucleating temperature (denoted as *T_SN_*, the lowest temperature within Domain Ⅱ is preferred) and kept for 5 min.
(d) Then, it was rapidly cooled to the predetermined crystallization temperature (*T_cSN_*) and held for a certain time to allow the completion of crystallization.
(e) Finally, the sample was heated to 200 °C at a rate of 10 °C/min.

#### 2.4.8. Polarized Optical Microscopy (POM)

The morphology of the samples was studied with a ZEISS MC-80 polarized light microscope (Carl Zeiss AG., Jena, Germany) equipped with a LINKAMTP-91 hot stage and a camera system. Thin melt-samples were prepared between microscope coverslips. Under a nitrogen atmosphere, they were melted at 200 °C for 5 min to erase the thermal history of the sample, and then they were rapidly cooled down to 130 °C for isothermal crystallization.

## 3. Results and Discussions

### 3.1. Chemical Structure of GO-H202

**Figure 1:**
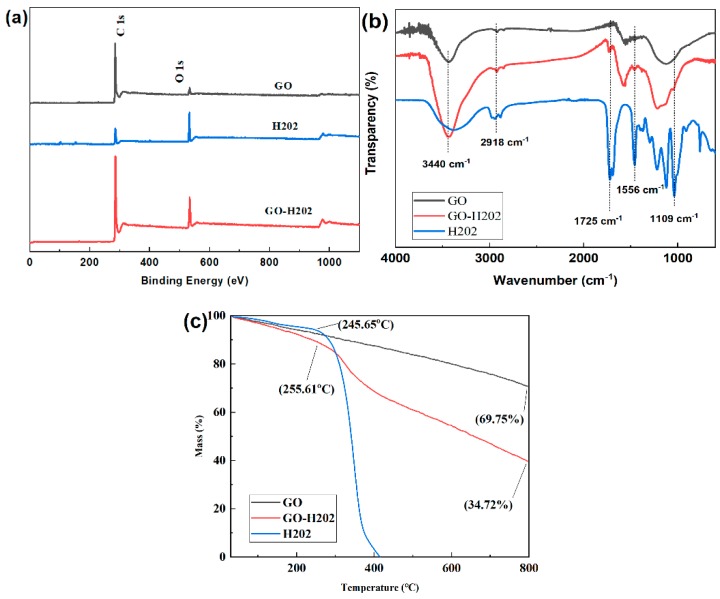
XPS (**a**), FT-IR spectrum (**b**) and TGA curves (**c**) of hyperbranched polyester H202, graphene oxide (GO), and GO-H202.

To investigate the chemical structure and constitution of H202, GO and GO-H202, the measurements of XPS, FT-IR, and TGA were performed, as shown in Figure 1. From Figure 1a, the XPS spectra and the fractions of O and C of the samples were calculated, as shown in Table 1.

Figure 1a and Table 1 reveal that after grafting H202, the O content of GO-H202 (27.7%) is obviously higher than that of GO (16.2%). The O/C ratio, *n*(O)/*n*(C), of GO-H202 is twice compared to GO, indicating that H202 has been successfully grafted onto GO.

On the other hand, Figure 1b showing the FT-IR reveals that for hyperbranched polyester H202, the wide peak at 3400 cm^−1^ represents the vibration of –OH group, while signals at the wavenumbers of 2918 cm^−1^ and 2888 cm^−1^ represent the asymmetric and symmetric vibrations of –CH_3_ and –CH_2_, respectively. The peak at the wavenumber of 1734 cm^−1^ is the stretching vibration peak of carbonyl from the polyester [35]. From the FT-IR spectrum of GO-H202, the extra peak at the wavenumber of 1734 cm^−1^ can be seen compared with that of neat GO, suggesting that H202 has been successfully grafted onto GO.

The TGA curves of H202, GO, and GO-H202 in Figure 1c show that the degradation of H202 initiates at about 245 °C and finishes at about 410 °C. Interestingly, a significant weight loss of GO-H202 is observed at the same temperature range, which cannot be observed from the TGA curve of neat GO, suggesting that the grafting of H202 onto GO is successful. Moreover, the grafting weight ratio of H202 is about 35 wt % of GO.

**Figure 2:**
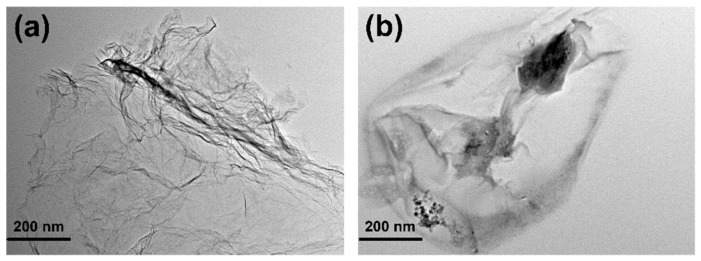
TEM images of (**a**) GO and (**b**) GO-H202.

To directly observe the morphology of the fillers, TEM was performed, as shown in Figure 2. Clearly, compared with neat GO, many dark dots can be observed from the TEM image of GO-H202, which might be evidence for the successful grafting of H202 onto GO.

### 3.2. DSC Cooling and Heating Behavior of the Composites

**Figure 3:**
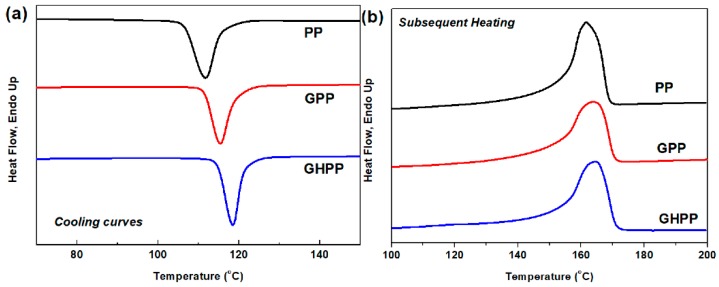
(**a**) Differential scanning calorimetry (DSC) cooling curves and (**b**) subsequent heating curves of the samples. The cooling and subsequent heating rates were 10 °C/min.

The DSC cooling and subsequent melting curves at the cooling and heating rates of 10 °C/min are shown in Figure 3, and the corresponding onset, endset and peak temperatures of crystallization (*T_conset_*, *T_cendset_* and *T_c_*); the peak, onset, and endset temperatures of melting (*T_m_*, *T_monset_* and *T_mendset_*); and the relative degree of crystallinity *X*_c_ are shown in Table 2.

As can be seen from Figure 3 and Table 2, compared with PP, the *T_c_*, *T_conset_*, and *T_cendset_* on the crystallization curve; the *T_m_*, *T_monset_*, and *T_mendset_* on the melting curve; and the relative degree of crystallinity *X*_c_ of GPP increase evidently, indicating an enhancement of nucleation rate and crystallizability of the composites after the addition of GO. Moreover, the lamellar thickness is enhanced as well. Interestingly, after the addition of GO-H202 (GHPP), these parameters further increase compared with GO, revealing the higher crystallization acceleration effect of GO-H202.

### 3.3. Isothermal Crystallization Kinetics of the Composites

**Figure 4:**
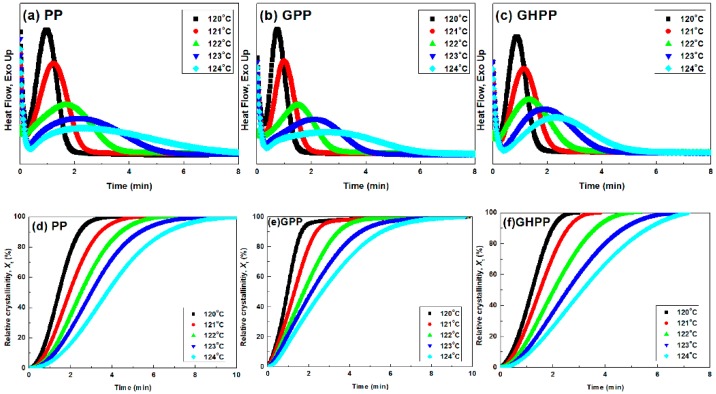
DSC isothermal crystallization curves and plots of relative crystallinity versus crystallization time for PP, GPP and GHPP at *T_cISO_* = 120–124 °C.

To further investigate the influence of GO and GO-H202 on the crystallization kinetics of the composites, the isothermal crystallization of the composites was investigated in this section. The isothermal crystallization curves and the relative degrees of crystallinity (*X*_t_) at the isothermal crystallization temperatures (*T_cISO_*) of 120–124 °C are shown in Figure 4 as a function of crystallization time. The corresponding exothermic DSC curves are also presented in Figure 4. The *X*_t_ is a relative value and could be defined as follows [54,55,56]:

(1) $$X_{t}=\int_{0}^{t}(dH/dt)dt/\int_{0}^{\infty }(dH/dt)dt$$

where d*H*_c_ denotes the measured enthalpy of crystallization during isothermal time interval d*t*. The limits *t* and *∞* denote the elapsed time during the crystallization and at the end of the crystallization process, respectively.

Figure 4 reveals that for all the samples, obvious dependence of the isothermal crystallization rate on crystallization temperature *T_cISO_* can be observed. As the supercooling (the difference between the melting and crystallization temperature) decreases, the exothermal peak becomes broader, indicating a decrease of crystallization rate as the *T_cISO_* increases.

The half crystallization time of overall crystallization (*t_o__0.5_*) is a direct measure of overall crystallization rate, which can be defined as the half period crystallization. The reciprocal of *t_o__0.5_*, represented by *G_o__0.5_* (Equation (2)) can be used as a parameter characterizing the crystallization rate of the sample.

(2) $$G_{o}{}_{0.5}=1/t_{o}{}_{0.5}$$

**Figure 5:**
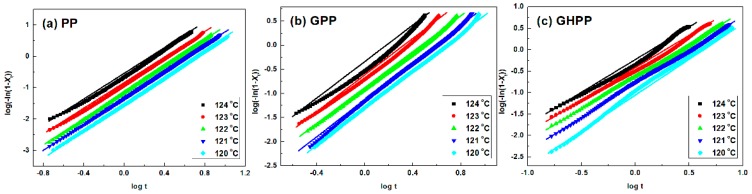
Avrami plots of log [−ln(1 − *X_t_*_)_] versus logt for isothermal crystallization process for (**a**) neat PP (**b**) GPP, and (**c**) GHPP.

Moreover, the Avrami model was employed to analyze the isothermal crystallization kinetics of the samples. The logarithmic form of Avrami model is expressed as Equation (3):

(3) $$In[-In(1-X_{t})]=Ink_{n}+nInt$$

where *X_t_* is relative degree of crystallinity at crystallization time *t*, *n* is the Avrami exponent, and *k_n_* represents the crystallization rate parameter involving both the nucleation and growth rate of crystals. By fitting the experimental data to Equation (3), the values of *n* and *k_n_* can be obtained from the slope and intercept of the plots of log [−ln(1 − *X_t_*)] versus log *t* for each cooling rate shown in Figure 5.

**Figure 6:**
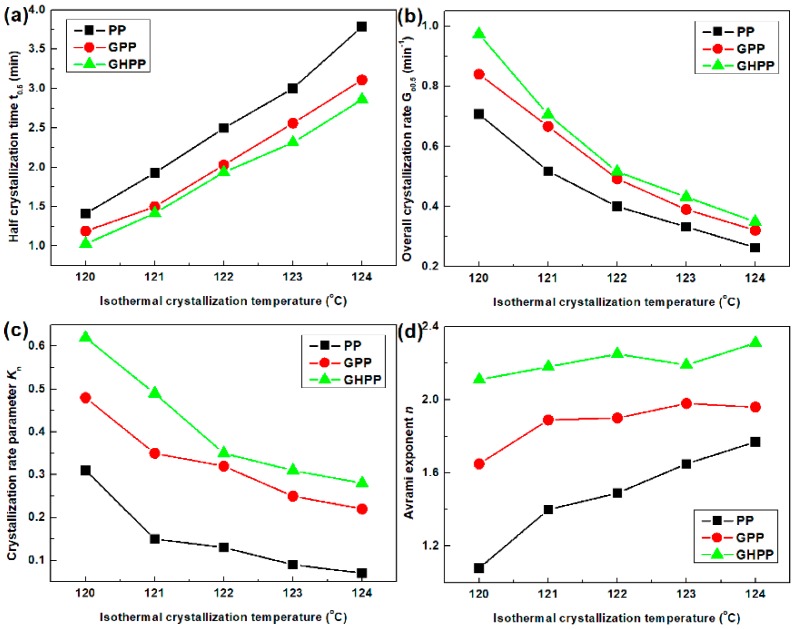
(**a**) Half crystallization time for overall crystallization, *t_o0.5_*, (**b**) overall crystallization rate parameter, *G_o0.5_*, (**c**) crystallization rate parameter involving both nucleation and crystal growth, *k_n_*, and (**d**) Avrami exponent *n* of samples as a function of isothermal crystallization temperature.

The *t_o0.5_*, *G_o0.5_*, *n*, and *k_n_* were calculated and plotted in Figure 6 as a function of crystallization temperature (*T_cISO_*).

It can be seen from Figure 6 that for all samples, *G_o0.5_* decreases gradually with an increase in the crystallization temperature *T_cISO_*. At a given *T_cISO_*, the *t_o0.5_* decreases gradually from PP, GPP to GHPP; meanwhile, both the *G_o0.5_* value and the kinetic parameter *k_n_* increase, indicating that the overall crystallization rate (involving both nucleation and crystal growth) increases after the addition of GO and GO-H202. After grafting hyperbranched polyester H202, the crystallization acceleration of GO-H202 becomes stronger compared with GO.

Moreover, the Avrami exponent *n* is broadly used to describe dimensionality. The regions from lower than 2, 2 to 3, and 3 to 4 correspond to one-, two-, and three-dimensional growth, respectively. As the crystallization temperature increases, the variations of Avrami exponent *n* of the three samples are quite different from each other. For neat PP, the Avrami exponent *n* strongly depends on the isothermal crystallization temperature *T_cISO_*, while less dependency can be observed for GPP. The *n* of neat PP and GPP are lower than two, corresponding to one-dimensional growth; for GHPP, *n* exhibits little dependency on the *T_cISO_*, which is higher than two, indicating that the addition of GO-H202 leads to two-dimensional growth during crystallization. Results above imply that the addition of GO and GO-H202 leads to different crystallization dimensionalities during the isothermal crystallization of PP.

### 3.4. Self-Nucleation Isothermal Crystallization Kinetics

**Figure 7:**
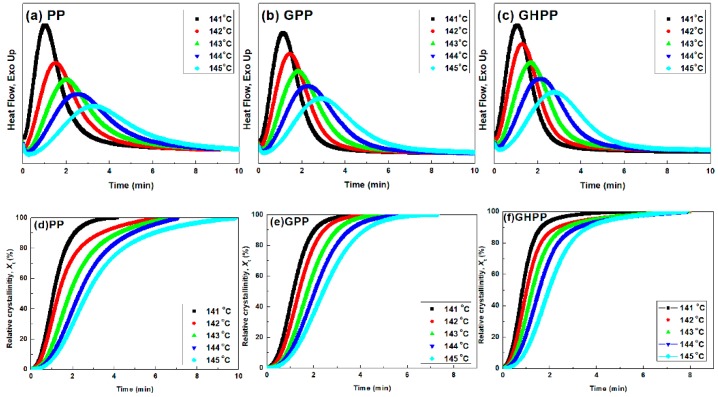
Self-nucleation isothermal crystallization curves (**a**) neat PP, (**b**) GPP and (**c**) GHPP and plots of relative crystallinity vs. crystallization time for (**d**) neat PP, (**e**) GPP and (**f**) GHPP when self-nucleated at 167 °C for 5 min and subsequently isothermally crystallized at *T_cSN_* = 141–145 °C.

In the isothermal crystallization study above, both primary nucleation and crystal growth contribute to the overall crystallization kinetics of PP. Unfortunately, the information about the single nucleation kinetics or the crystal growth kinetics can hardly be obtained. In order to separate the contributions of these two factors, the self-nucleation isothermal crystallization kinetics was carried out. It is assumed that the sample is fully nucleated during the self-nucleation process before the subsequent isothermal crystallization [49,57,58,59]. After that, the sample is rapidly cooled down and isothermally crystallized at the desired temperature (denoted as *T_cSN_*). In this way, the subsequent isothermal crystallization can be used to study the crystal growth kinetics. In this study, the temperature of self-nucleation treatment was 167 °C and the value of *T_cSN_* was 141–145 °C. The crystallization curves, as well as the curves of the relative degree of crystallinity (*X*_t_) as a function of crystallization time at *T_cSN_* are shown in Figure 7.

**Figure 8:**
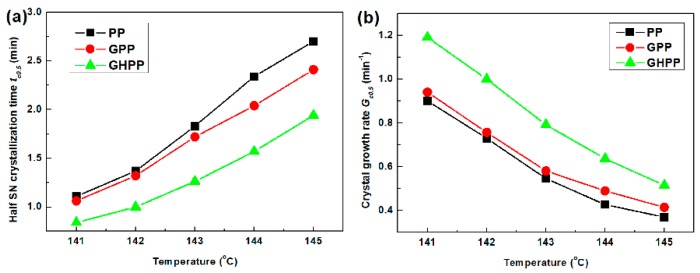
(**a**) Half crystallization time for crystal growth, *t_c0.5_*, and (**b**) Crystal growth rate parameter, *G_c0.5_*, as a function of crystallization temperature *T_cSN_* for PP, GPP and GHPP.

From Figure 7, the half-crystallization time for crystal growth, *t_c0.5_*, and the crystal growth rate parameter, *G_c0.5_*, were calculated and plotted in Figure 8 as a function of the crystallization temperature *T_cSN_*.

As can be seen from Figure 8, at the same *T_cSN_*, from PP, GPP to GHPP, *t_c0.5_* decreases monotonously while *G_c0.5_* increases gradually, indicating that the crystal growth rate of PP increases gradually after the addition of GO and GO-H202.

To understand fully, Hoffman and coworkers [60,61] proposed an equation based on the Turnbull and Fisher equation which is usually referred to as Lauritzen–Hoffman theory (Equation (4)):

(4) $$G=G_{0}\exp\left[\frac{-U^{*}}{R(T_{c}-T_{\infty })}\right]\exp\left[\frac{-K_{g}}{T_{c}(\Delta T)f}\right]$$

where *G* is the crystal growth rate, *G*_0_ is the constant and includes all the terms that are temperature-insensitive, U* is the transport activation energy, *R* is the gas constant, and *R* = 8.314 J/(mol·K), U^*^ = 6276 J/mol. *K_g_* is the nucleation parameter, *T_∞_* is the temperature below at which motion ceases and is usually taken as *T_∞_* = *T_g_* − 30K, *T_c_* is the crystallization temperature, $\Delta T=T_{m}{}^{0}-T_{c}$ is the degree of undercooling, *T_m_^0^* is the equilibrium melting temperature, and *f* is a factor that accounts for the variation in the enthalpy of fusion, *△h_f_*, with temperature, and is obtained by $f=2T_{c}/\left(T_{m}{}^{0}+T_{c}\right)$.

By rearranging Equation (4), Equation (5) is obtained:

(5) $$InG=InG_{0}-\left[\frac{U^{*}}{R\left(T_{c}-T_{\infty }\right)}\right]-\left[\frac{K_{g}}{T_{c}\cdot \Delta T\cdot f}\right]$$

*K_g_* is also expressed as Equation (6)

(6) $$K_{g}=n_{c}b_{0}\sigma \sigma_{e}T_{m}{}^{0}/\Delta h_{f}k$$

where the *n_c_* value depends on the crystallization regime according to Lauritzen–Hoffman theory. At Regime Ⅰ and Regime Ⅲ, which occur at low and high undercoolings, respectively, *n_c_* = 4. However, at Regime Ⅱ, which occurs at medium undercooling, *n_c_* = 2. *σ* and *σ_e_* are the lateral and end surface free energies of the growing crystal, respectively. *b*_0_ is the molecular thickness, *△h_f_* is the enthalpy of fusion, and *k* is the Boltzmann constant, *k* = 1.38 × 10^−23^ J/K. It has been reported that, for iPP resins, crystallization occurring at 139–154 °C was carried out in Regime Ⅱ. In this study, since the self-nucleation isothermal crystallization temperature is 141–145 °C, *n_c_* is taken to be 2 [62,63].

*σ* can also be estimated as:

(7) $$\sigma =\alpha \Delta h_{f}\sqrt{a_{0}b_{0}}$$

where *α* was empirically obtained to be 0.1 and *a*_0_*b*_0_ represents the cross-sectional area of the polymer chain [60]. *△h_f_*, *a_0_*, and *b*_0_ of iPP are supposed to be 1.96 × 10^8^ J/m^3^, 5.49 × 10^−10^ m, and 6.26 × 10^−10^ m, respectively, based on the literature [60]. Therefore, a value of *σ* = 11.5 erg cm^−2^ is obtained from Equation (7).

**Figure 9:**
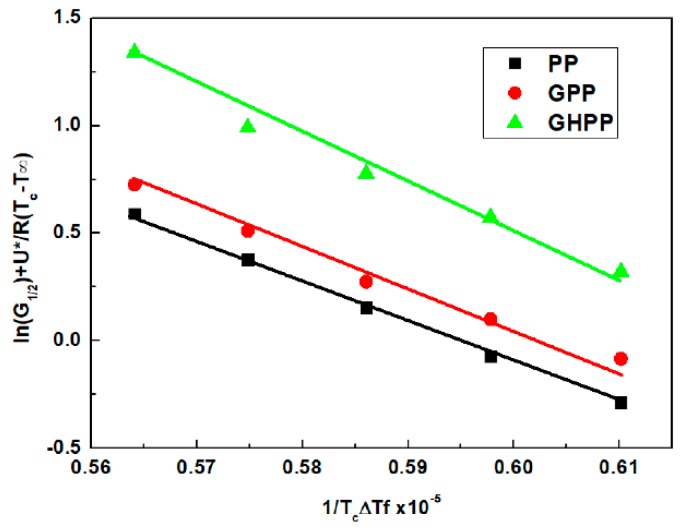
Lauritzen–Hoffman kinetic theory plot for PP, GPP, and GHPP.

From the plot of $InG+\frac{U^{*}}{R\left(T_{c}-T_{\infty }\right)}$ of against $\frac{1}{T_{c}\cdot \Delta T\cdot f}$ (shown in Figure 9), the value of *K_g_* can be directly calculated from the slope. Therefore, the value of *σ_e_* was simply estimated by Equations (5) and (6). The results of *K_g_* and *σ_e_* for samples are listed in Table 3.

The results in Table 3 suggest that, from PP, GPP to GHPP, both the *K_g_* and *σ_e_* decrease gradually, indicating that the surface free energy for crystal growth decreases and the ability for crystal growth increases after the addition of GO and GO-H202. The addition of GO might facilitate the molecular chain regularly arranging into lamellae to a larger degree and therefore increases the growth rate of the crystal. Moreover, the addition of GO-H202 further increases the crystal growth rate of PP benefit from the grafting of H202 on GO, which might make the GO’s dispersion more uniform and act as a foreign nucleating agent for iPP.

In summary, the crystallization kinetics analysis of this manuscript demonstrates that the presence of GO not only enhances the nucleation rate and overall crystallization rate, but also encourages the crystal growth of the PP matrix, possibly due to the external nucleating agent effect of GO; moreover, the grafting of hyperbranched polyester onto GO provides a better compatibility (i.e., better dispersion) with iPP matrix and possibly new nucleation sites for iPP, resulting in the further enhancement of both nucleation rate, crystal growth rate, and overall crystallization rate.

### 3.5. Polarized Optical Microscopy Observation

The overall crystallization kinetics and crystal growth kinetics of the composites were obtained using DSC method above. However, the impact of GO and GO-H202 on the crystalline morphology of the samples was still unknown. To understand fully, polarized optical microscopy obervation during isothermal crystallization process was performed. The samples were heated at 200 °C for 5 min and then rapidly cooled to 130 °C for isothermal crystallization.

**Figure 10:**
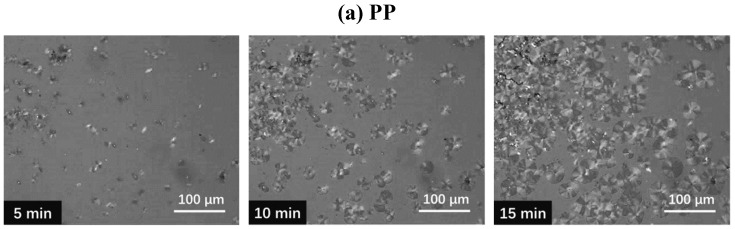

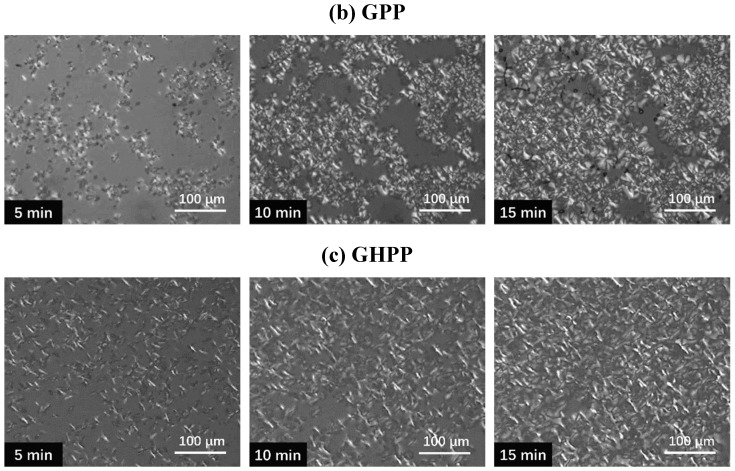
Polarized optical microscopy (POM) images during the isothermal crystallization at 130 °C of (**a**) neat PP, (**b**) GPP, and (**c**) GHPP.

Figure 10 shows the morphological evolution of the samples during the isothermal crystallization at 130 °C. It can be seen that at 5 min after crystallization began, for neat PP, some sporadic nuclei are visible, while for GPP and GHPP, much more amounts of nuclei can be seen, indicating that after the addition of GO or GO-H202, the nucleation rate of the composites is evidently enhanced, which is in accordance with the DSC analysis above; as the crystallization time increases, at 10 min, it can be observed that the spherulites grow continuously; at 15 min, GHPP finished crystallization, and the crystallites occupied the whole image, while for neat PP and GPP, the crystallization was not finished yet.

Interestingly, the shape of the crystallites of GHPP is quite different from GPP and neat PP. Considering that the Avrami exponent *n* changed after the addition of GO-H202 (Figure 6d), it is proposed that the addition of GO-H202 might change the nucleation and crystal growth manner and further change the crystallite morphology of the composites.

## 4. Conclusions

In this study, we prepared the hyperbranched polyester grafted GO (GO-H202) and the iPP/GO composites. Results of XPS, FT-IR, and TEM revealed the successful grafting of H202 onto GO, and TGA indicated that the weight ratio of grafting was about 35 wt %. DSC analysis and POM observation were carried out to investigate the role of GO and GO-H202 on the crystallization kinetics of the composites. Results of conventional cooling and heating scan suggested that the addition of GO enhances the nucleation rate and crystallizability of the composites, while GO-H202 exhibits a higher crystallization acceleration effect compared with GO. Results of isothermal crystallization kinetics and self-nucleation isothermal crystallization kinetics showed that the overall crystallization rate (involving both nucleation and crystal growth) and crystal growth rate increase after the addition of GO and GO-H202. The crystallization acceleration of GO-H202 became stronger compared with GO. Moreover, the variation trends of Avrami exponent *n* with the isothermal crystallization temperature *T_cISO_* changed significantly from PP, GPP to GHPP, which might imply that the addition of GO and GO-H202 led to different crystallization dimensionalities during the isothermal crystallization of the composites.

## Figures and Tables

**Table 1:** Fractions of C and O of H202, GO, and GO-H202.

Samples	O 1s (%)	C 1s (%)	*n*(O)/*n*(C)
H202	60.5	39.5	1.53
GO	16.2	83.8	0.19
GO-H202	27.7	72.3	0.38

**Table 2:** Crystallization and melting parameters of polypropylene (PP), iPP/GO (GPP), and iPP/GO-H202 (GHPP).

Sample	Cooling Scan				Subsequent Heating Scan			
	*T_c_* (°C)	*T_conset_* (°C)	*T_cendset_* (°C)	Width (°C)	*T_m_* (°C)	*T_monset_* (°C)	*T_mendset_* (°C)	*X_c_* (%)
PP	111.7	118.5	106.5	12.0	161.5	153.1	166.0	48.6
GPP	115.4	121.0	110.7	10.3	163.6	153.9	167.2	49.5
GHPP	118.5	122.6	114.4	8.2	164.8	155.2	168.7	50.9

**Table 3:** The kinetic parameter for crystal growth (*K_g_*) and interfacial free energies of crystal growth (*σ_e_*) under self-nucleation isothermal crystallization process.

Sample	*K_g_* × 10^−4^ (K^2^)	*σ_e_* (erg cm^−2^)
PP	23.4	96.6
GPP	20.2	83.1
GHPP	17.9	76.0
